# Effects of Aerobic Fitness on Aging-Related Changes of Interhemispheric Inhibition and Motor Performance

**DOI:** 10.3389/fnagi.2013.00066

**Published:** 2013-10-30

**Authors:** Keith M. McGregor, Joe R. Nocera, Atchar Sudhyadhom, Carolynn Patten, Todd M. Manini, Jeffrey A. Kleim, Bruce Crosson, Andrew J. Butler

**Affiliations:** ^1^Center for Visual and Neurocognitive Rehabilitation, U.S. Department of Veterans Affairs, Decatur, GA, USA; ^2^Department of Neurology, Emory University, Atlanta, GA, USA; ^3^VA Center of Excellence, Gainesville, FL, USA; ^4^Department of Neurosurgery, University of Florida, Gainesville, FL, USA; ^5^Department of Physical Therapy, University of Florida, Gainesville, FL, USA; ^6^Department of Geriatrics, University of Florida, Gainesville, FL, USA; ^7^School of Biological and Health Systems Engineering, Arizona State University, Tempe, AZ, USA; ^8^Department of Physical Therapy, Georgia State University, Atlanta, GA, USA

**Keywords:** aging neuroscience, aging, fMRI, TMS, physical fitness, interhemispheric communication, negative BOLD, dexterity

## Abstract

Physical fitness has been long associated with maintenance and improvement of motor performance as we age. In particular, measures of psychomotor speed and motor dexterity tend to be higher in physically fit aging adults as compared to their sedentary counterparts. Using functional magnetic resonance imaging (fMRI) and transcranial magnetic stimulation (TMS), we explored the patterns of neural activity that may, in part, account for differences between individuals of varying physical fitness levels. In this study, we enrolled both sedentary and physically fit middle age (40–60) and younger (18–30) adults and measured upper extremity motor performance during behavioral testing. In a follow-up session, we employed TMS and fMRI to assess levels of interhemispheric communication during unimanual tasks. Results show that increased physical fitness is associated with better upper extremity motor performance on distal dexterity assessments and increased levels of interhemispheric inhibition in middle age adults. Further, the functional correlates of changes of ipsilateral activity appears to be restricted to the aging process as younger adults of varying fitness levels do not differ in hemispheric patterns of activity or motor performance. We conclude that sedentary aging confers a loss of interhemispheric inhibition that is deleterious to some aspects of motor function, as early as midlife, but these changes can be mediated by chronic engagement in aerobic exercise.

## Introduction

As we age, manual dexterity and upper extremity motor performance begins to decline. While there are many factors that are implicated in this process from a mechanical level (e.g., rheumatism, decreased muscle mass, increased rigidity of connective tissues) the predominant factor for aging-related decreases in hand motor function is most likely caused by alteration of neural function within the central nervous system (Cole et al., 1998; Latash and Zatsiorsky, 2009; Christou, 2011). There is long-standing evidence that indicates that the continuous engagement in aerobic activity throughout the lifespan helps maintain dexterity and coordinated hand function (Spirduso, 1975, 1980; Spirduso et al., 1988). However, the link between neural function, aerobic activity, and measures of motor performance in aging has only recently been approached. Application of functional magnetic resonance imaging (fMRI) and transcranial magnetic stimulation (TMS) to address this problem has discerned that aging-related changes in patterns of cortical activity predicts changes in motor function associated with aging (Fling et al., 2011a; McGregor et al., 2011, 2012a; Bernard and Seidler, 2012). Moreover, these aging-related changes in activity may be altered by the regular engagement in aerobic exercise, which may explain the maintenance of hand dexterity in exercising aging adults (McGregor et al., 2011, 2012b; Davidson and Tremblay, 2013).

An increasing body of literature indicates that as we age, we experience a decrease in the level of interhemispheric inhibition in primary sensorimotor areas particularly during execution of motor tasks in the upper extremity (Sale and Semmler, 2005; Riecker et al., 2006; Fujiyama et al., 2009; Seidler et al., 2010; McGregor et al., 2011; Petitjean and Ko, 2012; Davidson and Tremblay, 2013). During unimanual hand movements the contralateral (to the moving hand) primary motor cortex results in increased metabolic activity and increased activity relative to baseline resting conditions (Rao et al., 1993; Allison et al., 2000; Newton et al., 2005). However, recent evidence has revealed that activity of the ipsilateral cortex during such movements may vary according to one’s chronological age or level of physical conditioning (McGregor et al., 2011, 2012a; Davidson and Tremblay, 2013). The presence of interhemispheric inhibition can be measured using fMRI, where during unimanual tasks, activity in the ipsilateral primary motor cortex (iM1) is suppressed or deactivated, as indicated by a negative BOLD response (decreased T2* signal to levels below that of resting baseline conditions) in this area (Allison et al., 2000; Stefanovic et al., 2004; Newton et al., 2005; Riecker et al., 2006; McGregor et al., 2009, 2011). This signal pattern is most prevalent in younger adults (18–20 years) (Riecker et al., 2006). In contrast, during similar movements, elderly adults (60+ years) appear to recruit the ipsilateral motor (iM1) cortex, indicated by the presence of a positive BOLD response in this area (Hlushchuk and Hari, 2006; Naccarato et al., 2006; Riecker et al., 2006; Ward et al., 2008; McGregor et al., 2009, 2011; Langan et al., 2010).

However, the recruitment of the ipsilateral cortex in elderly adults may vary as a function of their physical fitness level. Recently, Voelcker-Rehage et al. (2010) reported that elderly adults (mean: 68 years) with higher levels of motor (muscle strength and coordination) and physical fitness, evidenced decreases in ipsilateral BA4 activity relative to baseline during a unimanual button response task. In our own lab, we compared elderly adults (60+ years) who regularly engaged in aerobic physical activity against a relatively sedentary elderly cohort using both fMRI and TMS to assess interhemispheric inhibition. The results showed that not only were the aerobically active elderly adults more likely to show a negative BOLD in fMRI in the ipsilateral motor cortex, but these individuals also evidenced significantly longer ipsilateral silent periods (iSPs) (a measure of interhemispheric inhibition), as assessed by TMS (McGregor et al., 2011). A recent study with elderly adults by Davidson and Tremblay (2013) also yielded similar findings respective of TMS in that the most physically fit individuals showed the longest iSPs. Based on the above evidence, there is sufficient support to contend that as we enter sedentary senescence (60+ years), interhemispheric inhibition decreases and may be correlated to changes in motor function.

However, aging is not a discrete process and the selection of only extreme age cohorts (18–30 or 60+ years) represents a challenge for cross-sectional investigation relating to neural control of movement. What happens in middle age strongly informs on the processes of change that appear in senescence. To address this concern, the present investigation enrolled middle age adults (40–60 years) of varying fitness levels for assessment of interhemispheric communication. We contrast this age cohort with younger adults (18–30 years), also of varying fitness level. As such, the present study is a cross-section of middle age (*N* = 38) and younger adults (*N* = 21), each grouped by their self-reported (validated by a direct aerobic assessment) aerobic activity. We employed both fMRI and TMS with these individuals to evaluate levels of interhemispheric inhibition using each modality in addition to examining each participant’s performance on a battery of motor assessments. In fMRI, participants engaged in a simple unimanual tapping sequence previously shown to evoke differential BOLD response across age group comparisons (McGregor et al., 2011). For TMS, we assessed each participant’s iSP, which is an index of interhemispheric inhibition (Meyer et al., 1995). An additional focus of the present study was to investigate the relationship of motor function tests to BOLD and TMS indices of interhemispheric inhibition. We hypothesized that more physically active individuals would show greater levels of interhemispheric inhibition and better motor performance. Further, we hypothesized this trend would be exacerbated in comparisons of middle age adults. We believe this project is the first study to apply both neuroimaging and neurophysiological techniques to probe the effects of cardiovascular fitness on the relationship between motor function and aging in midlife.

## Materials and Methods

### Participants

Of 240 screened candidates we enrolled 38 middle aged (ages 40–60) and 21 (ages 19–32) younger adults in the current study who were reportedly healthy at the time of screening and study participation. Individuals were screened into one of two categories, either sedentary or physically active, based on self-reported exercise activity (Physical Activity Readiness Questionnaire – PARQ) and performance on a submaximal cardiovascular fitness assessment (YMCA Cycling Protocol). Eight additional participants (not included in the above total) were removed from study consideration due to mismatch fitness screen and self-report, scheduling difficulties, or reported discomfort in a mock MRI environment. Included participant characteristics are summarized in Table 1. All participants were right handed as assessed by the Edinburgh Handedness Inventory (Oldfield, 1971). Participants qualifying for the sedentary group were comprised of reportedly healthy individuals that engaged in voluntary cardiovascular exercise for fewer than 90 total minutes per week and had an assessed VO_2_max estimate of <35. This group was termed the “sedentary group” for each age cohort. The second group of adults was comprised of healthy individuals who reportedly engaged in bouts of medium to vigorous voluntary aerobic (swimming, bicycling, jogging, etc.) exercise lasting at least 45 min at least three times per week and an assessed estimate of VO_2_max of >35. As such, there were four groups in the current study: physically active middle age adults, sedentary middle age adults, physically active younger adults, and sedentary younger adults. Of the physically active middle age adults, 12 individuals reported they engaged in physically active professions (e.g., personal trainer, aerobic fitness instructor).

**Table 1:** **Overall participant characteristics**.

	Middle age	Younger
Age*	52.1 (6.44) (41–60)*	22.1 (3.41) (19–29)*
*N*/gender	38/21 Female	21/12 Female
Education	16.1 (2.33)	15.6 (1.9)
BMI*	24.4 (3.9)	24.36 (5.11)
VO_2_max	38.76 (14.1)	40.25 (11.37)
Weekly activity (min)*	76 (33.4)	64.7 (32.5)

BMI, body mass index. Cell values denote group means. Parentheses indicate standard deviation within cell. Bracket indicates range. Student’s *t*-test contrast significance at *p < 0.05.

We excluded individuals with cardiac history (angina, prior cardiac arrest, uncontrolled hypertension) contraindicating tests of aerobic fitness. We also excluded individuals with contraindications to MRI including metal implants, alcohol, or drug abuse, neurological disorder (tremor, stroke, motor disorders, multiple sclerosis), major psychological disorder, or hearing difficulties. A recent medication history was taken for each participant. Persons on medications that were contraindicated for fitness testing (beta blockers) were also disqualified from the study. Cortical activation characteristics in primary motor cortex may be altered by skilled (Jancke et al., 2000; Krampe et al., 2002) or repeated digit movement practice (Gordon et al., 2008) so we excluded participants if they reported upon inquiry that they engaged in repeated skilled finger practice (musical instruments, expert typing, etc.) with practice specified as at least three 1-h training sessions performed on a weekly basis. Study personnel completed the informed consent process with each participant following protocols approved by the University of Florida’s Institutional Review Board (IRB).

### Sessions and procedures

**Figure 1:**
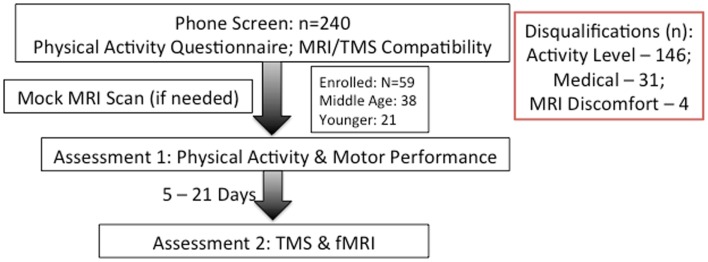
**Flowchart of study**. Category and number of screen-outs are listed on right.

Participation for the study occurred over two sessions separated by at least five, but no more than 21 calendar days. We attempted to schedule both sessions at the same time of day to avert potential differences due to circadian cycles. Participants were asked to refrain from drinking caffeinated beverages at least 3 h prior to each session to prevent potential signal alteration in imaging. Figure 1 presents a flowchart of study participation.

### First session: Motor assessment and fitness measures

#### Motor assessment

Participants completed motor assessments of the dominant hand including: hand and pinch grip strength, the Halstead Finger Tapping task, simple reaction time assessment, Purdue pegboard (pin placement and assembly tasks), Poffenberger Crossed-Uncrossed difference test, hand steadiness test (Lafayette Instruments, Lafayette, IN, USA), and the Nine-Hole Pegboard task. Additionally, to test distal motor dexterity, participants engaged in a coin-rotation task with two conditions. In the first condition, the participant rotated a coin (U.S. nickel) 20 times as quickly as possible using the index finger, middle finger, and thumb, with time to completion as the outcome measure. An assessment of motor dysfunction in neurological practice, the coin-rotation test has been shown to be diagnostic of distal motor function both in cases of suspected pathology and aging in the absence of pathology (Hanna-Pladdy et al., 2002; Mendoza et al., 2009). In the second coin-rotation condition, the participant maintained an isometric pinch force of 25–35% of maximum voluntary force with a pinch grip dynamometer using a lateral grip. Coin-rotation tasks were performed with both the left and right hands. Both the hand used for coin-rotation and trial condition (unimanual or bimanual task) were pseudo-randomized and counter-balanced across participants to account for potential order effects. Accidental coin drops were excluded from consideration and the participant repeated the trial if a drop occurred. Participants were allowed three to five practice trials to acclimate to the rotation task in each task condition. The difference score between the bimanual and unimanual task conditions was calculated to assess the effect of bimanual activity. Grip force was measured in lateral pinch, precision pinch, and power grip using Jamar brand dynamometers. For the simple reaction time test and crossed-uncrossed difference test, E-Prime software (PST Software, Pittsburgh, PA, USA) was used to present a target stimulus on a computer screen situated 60 cm away from the participant’s chin. In both paradigms, participant maintained surface contact with the hand and depressed a computer keypad button as quickly as possible in response to the visual stimulus. Inter-stimulus intervals were pseudo-randomized between 250 and 1000 ms. The simple reaction time task involved the presentation of a stimulus in the center of the screen and the participant response only with the right hand for 50 trials. The Poffenberger Crossed-Uncrossed difference test required the participant to visually fixate at a centered fixation cross and make an immediate keyboard response when a small circle was presented offset 3° either to the right or left of the fixation cross (Marzi, 1999). This was done in consecutive blocks of 100 trials for the right and left hand, respectively. The uncrossed response was the time required to press the button when the circle was presented in the same visual hemifield as the hand responding (e.g., right side stimulus with right-hand response). The crossed response condition was the time required to press the button when the stimulus was in the opposite visual hemifield (e.g., left side stimulus with right-hand response). The difference score between the two conditions has been shown to describe potential alteration of callosal function across age groups in previous work (Schulte et al., 2005), see also (Bernard et al., 2011).

#### Biometrics

After behavioral testing, the participant was brought to a physical assessment facility. There we compiled a list of physical function measures for each individual. These included height, weight, blood pressure, resting heart rate, and a simple test of balance (sitting at rest on the bicycle ergometer) to ensure compatibility with a cycle-based fitness assessment.

#### Aerobic endurance

To assess aerobic fitness level, we employed the cycle-based YMCA submaximal VO_2_ cardiovascular fitness assessment. This test is an accurate method to assess aerobic fitness level without placing maximal strain on the participant (Garatachea et al., 2007). In the YMCA test, heart rate workload values are obtained at two to four points and extrapolated to predict workload at the estimated maximum heart rate (MHR) (e.g., 220-age). VO_2_max is then calculated from the predicted maximum workload. Participants rode a stationary bicycle for two to four 3-min stages. The first stage was a warm-up at 50 revolutions per minute (RPM) at a power level of 25 W. During all testing stages, heart rate was continuously monitored to ensure the participant did not exceed 85% of age-predicted MHR, at which point the exam would be stopped. For the analysis, average heart rate during the final 30 s of the second and third minutes was plotted against workload for each stage. Three-minute trial workloads below were chosen based on the participants’ heart rate at the end of the warm-up period. The fourth 3-min stage was a cool-down period added to the end of the test. Outcome measures for the YMCA test were estimated VO_2_max and estimated liters of oxygen consumed per minute.

### Second session: Transcranial magnetic stimulation and functional MRI

After at least five calendar days, the participant was scheduled for the second and final participation session. This session lasted for about 3 h and involved single-pulse TMS followed by fMRI.

### Single-pulse transcranial magnetic stimulation

For the TMS procedure, participants sat in a comfortable chair within the confines of a stereotactic positioning frame. Full procedural details have been described in another article (Kleim et al., 2007). Electromyography (EMG) was taken from the first dorsal interosseous (FDI) muscle on both hands. Muscle activation was monitored with a real-time oscilloscope software package (LabChart 7.0, ADInstruments Ltd., Colorado Springs, CO, USA). A Magstim 200^2^ magnetic stimulator (The Magstim Company Ltd., Carmarthenshire, UK) with a 70-mm figure-of-eight coil was used to stimulate the left primary motor cortex during all TMS procedures. Navigation of the stimulator coil to target cortex was accomplished using coil registration to a standardized brain image provided by BrainSight software (Rogue-Research, Montreal, QC, Canada). During stimulation, the coil was placed tangential to the scalp with the handle pointing backwards and 45° away from the midline. The scalp site (“hotspot”) corresponding to the lowest stimulator output sufficient to generate a magnetic evoked potential (MEP) of at least 50 mV in 6 out of 10 trials was defined as the site of lowest motor threshold (LMT). This hotspot was the site of stimulation for all measures in the current investigation, which are described below.

Two single-pulse TMS measures were considered for the current study: LMT value and iSP. LMT value simply represents the stimulator output value as a percentage of maximum stimulator output. For the iSP assessment, the left FDI muscle was contracted via pinch grip at 20–30% maximal voluntary contraction (MVC), determined by pinch dynamometer, and a 150% LMT stimulus was delivered to the left primary motor area FDI hotspot. Twenty consecutive trials were performed for iSP assessments. Participants were allowed a brief (1–2 min) rest after every five trials to allay potential fatigue. Prior to silent period assessment, the participant was instructed to maintain the non-active hand in a prone, resting position. EMG of the contralateral hand was monitored for mirror activity and the trial was discarded and repeated if such activity was observed.

### Functional imaging

After the TMS session, participants were given a rest of ∼30 min during which time they were instructed on the procedures to be carried out during MRI. After acknowledging understanding of the tasks, the participant was brought to the imaging facility.

#### Parameters

Magnetic resonance images were acquired on a 3-T Achieva Whole-Body Scanner (Philips) using a 32-channel SENSE head coil. Head motion was minimized using foam padding and laser grid alignment. Before functional imaging sequences, structural images were acquired [160 mm × 1.0 mm thick sagittal slices, using a 3D T1-weighted sequence: time of echo (TE) = 8.057 ms; time of repetition (TR) = 3.685 ms; flip angle (FA) = 8]. Whole-brain high-resolution echo planar functional images (EPI) were acquired using 57 mm × 2 mm thick axial slices and the following parameters: TE = 30 ms; TR = 4000 ms; FA = 87; FOV = 192 mm × 192 mm × 114mm; Matrix = 96 × 96; SENSE factor = 1.5. Two dummy EPI acquisitions were acquired and discarded to allow for signal equilibration.

Stimuli were presented on a first surface mirror presentation system situated at the rear bore aperture of the magnet. Stimuli were sent via personal computer (PC) to a 30′′ high-resolution (2560 × 1600 pixels) MR-compatible LCD display (Philips *in vivo* Systems) via fiber optic connection. A large mirror reflected the LCD display into the bore of the magnet. A mirror situated on the head coil then reversed the mirror image for presentation to the participant.

#### fMRI task

A block-design, right-hand motor task was used to evaluate interhemispheric cortical activation patterns. Blocks consisted of seven images (28 s) for both rest and active conditions. Six cycles (alternating between seven rest images and seven active images) comprised each run (5 min 36 s). In the scanner, participants engaged in two runs of the motor task and all performance data (accuracy, reaction times) was saved for later analysis. Participants were trained on the task inside the scanner prior to data acquisition. Between runs, participants again verified their understanding of the task via verbal report.

The motor task was a block presentation of a repeated button squeeze using an index finger to thumb opposition (“button tapping”). This task has been shown to exhibit a negative BOLD response in ipsilateral primary motor cortex (M1) in young adults (Allison et al., 2000; Riecker et al., 2006; McGregor et al., 2011). Performance of similar tasks in sedentary elderly samples, however, shows positive BOLD responses in ipsilateral M1 (McGregor et al., 2009, 2011). Stimuli were presented using E-Prime software (PST Software, Pittsburgh, PA, USA). Button responses were made on a RP04U button response unit (BRU) manufactured by MagConcept (Sunnyvale, CA, USA) connected to the presentation computer. Researchers positioned the participants’ fingers in the correct posture on the BRU prior to acquisition and instructed on target force output. The participant was asked to use the index finger-thumb squeeze to depress a button for each trial press with only as much force as required to generate a tactile “click” on the response device (equivalent to roughly 3N). The participants’ left (non-active) hand was placed in a prone, resting position along the side of the body. Participants were visually monitored for movements of this hand during active task blocks. Consistent overt mirrored movement was used as a criterion for exclusion.

During the functional run, participants fixated gaze on a central fixation cross of a computer screen throughout each of two runs. Blocks were cued by the change of fixation cross varying between the word “Squeeze” (movement condition) or the word “Rest” (rest condition). During the movement condition, participants were instructed to time button presses with the flashing visual stimulus (2 Hz). Trials were briefly practiced in the scanner prior to image acquisition. Researchers in the scanner operation room monitored subject performance during the task.

### Analysis

#### Behavioral measures

##### Motor performance

Group data for behavioral measures were compared using between-subjects Student’s *t*-test and *p*-values ≤ 0.05 were considered statistically significant. Statistical analyses were completed using the application JMP 9.0 (SAS Institute, Cary, NC, USA), unless otherwise specified.

##### Physical activity

We measured physical activity based on estimated VO_2_max and self-reported weekly activity surveys. All individuals in the aerobically fit group had to exhibit a VO_2_max of 35 ml/min/kg or greater and report a weekly engagement in aerobic activity of over 135 min. Sedentary individuals had to exhibit an assessed VO_2_max of 34 ml/min/kg or lower and <90 min of physical activity per week. Tables 2–4 present group data of physical attributes.

**Table 2:** **Aerobically fit participant characteristics**.

	Active middle age	Active younger
Age*	51.3 (5.9) (41–60)*	23.1 (3.8) (19–29)*
*N*/gender	17/6 Female	12/6 Female
Education	16.1 (2.25)	16.4 (2.38)
BMI	22.1 (2.5)*	22.3 (3.5)
VO_2_max	49.8 (12.7)*	47.3 (9.8)
Weekly activity (min)	146 (23.4)*	126.7 (22.5)

BMI, body mass index. Cell values denote group means. Parentheses indicate standard deviation within cell. Bracket indicates range. Student’s *t*-test contrast significance at *p < 0.05.

**Table 3:** **Sedentary participant characteristics**.

	Sedentary middle age	Sedentary younger
Age	51.3 (5.9) (41–60)	22.4 (2.9) (19–28)
*N*/Gender	21/12 Female	9/6 Female
Education	16.1 (2.25)	14.4 (3.38)
BMI	26.1 (3.95)*	28 (5.65)
VO_2_max	29.8 (12.7)	30.8 (3.77)
Weekly activity (min)	36.7 (12.5)	40.3 (22.5)

BMI, body mass index. Cell values denote group means. Parentheses indicate standard deviation within cell. Bracket indicates range. Student’s *t*-test contrast significance at *p < 0.05.

**Table 4:** **Fitness group differences**.

	Middle age fit vs. sed	Younger fit vs. sed
Age	*t*(36) = 0.64, ns	*t*(19) = 0.4, ns
Education	*t*(36) = 1.47, ns	*t*(19) = 1.4, ns
BMI	*t*(36) = 3.51, *p* < 0.01*	*t*(19) = 2.7 *p* = 0.01
VO_2_max	*t*(36) = 6.22, *p* < 0.01*	*t*(19) = 5.3 *p* < 0.01
Weekly activity (min)	*t*(36) = 7.4, *p* < 0.01*	*t*(19) = 4.9 *p* < 0.01

BMI, body mass index. Cell values denote group means. Parentheses indicate standard deviation within cell. Bracket indicates range. Student’s *t*-test contrast significance at *p < 0.05.

##### Mirror movements

As a criterion for data exclusion, we also included the presence of mirror movements during unimanual muscle contraction at the TMS session. We assessed such movements during 20 unimanual contractions each of both the left FDI muscle (via submaximal force pinch grip) using EMG. A mirror score for each hand was calculated for each trial by taking the root mean square of the EMG in the mirror hand and dividing by the median EMG root mean square value (over the 20 contractions) in the voluntary hand. This procedure was adapted from previously reported work (Hermsdorfer et al., 1995; Verstynen et al., 2007; McGregor et al., 2011). The mirror scores for the trials were then averaged within each hand squeeze condition. Individuals exhibiting a mirror score >0.20 were to be removed from study consideration. No participants evidenced significant mirror scores over threshold, however.

#### Transcranial magnetic stimulation

**Figure 2:**
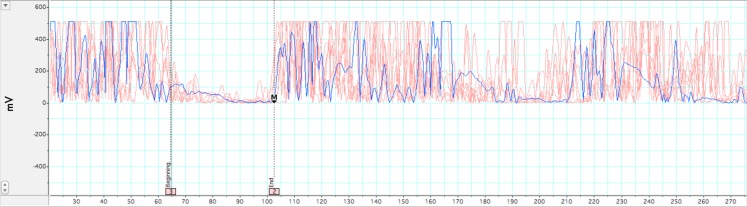
**Illustration of ipsilateral silent period**. Rectified EMG across multiple trials within a single participant while holding isometric force at 35–50% MVC. In-graphic lines represent beginning and end of EMG depression for the iSP. A pre-stimulus acquisition of 20 ms was acquired for baseline comparison. Motor evoked potential and subsequent silent period occurs at ∼38 ms. iSP duration for this participant was 42 ms.

Two measures were analyzed from TMS: LMT, duration of iSP. LabChart 7.0, JMP 9.0, and Microsoft Excel 2007 software were used to complete this analysis. The analysis of silent period duration was adapted from (Garvey et al., 2001). All EMG data was rectified and normalized to baseline of pre-stimulus EMG prior to analysis. EMG baseline was taken as mean of the 20-ms pre-stimulus waveform during pinchgrip. The latency of MEPs was measured from the onset of the stimulus presentation to the onset of the MEP. The first of five consecutive datapoints after MEP that evidenced a minimum decrease of 80% from mean EMG values from the 20-ms pre-stimulus recording period were taken as the silent period onset. Conversely, the first of five consecutive data points evidencing a return to >20% of pre-stimulus mean levels was set as the point of termination of the silent period. A sample silent period grouping for a subject session is presented in Figure 2. Group comparisons of TMS measures were analyzed using between-subjects Student’s *t*-test and *p*-values < 0.05 were considered statistically significant.

#### Functional magnetic resonance imaging

Functional images were analyzed and overlaid onto structural images with the Analysis of Functional Neuroimaging (AFNI) program (Cox, 1996). To mitigate spatial deviation from the structural to functional images, a local Pearson correlation registration procedure was implemented with AFNI’s 3dAllineate program (Saad et al., 2009). To minimize the effects of head motion, time series images were spatially registered in three-dimensional space to the first functional image using a linear rigid-body transform as implemented by AFNI’s 3dvolreg. A subject’s data was excluded from further analyses if any time series of a subject is judged, from visual inspection, to contain a significant number of images with gross artifacts or residual motion. However, sub-threshold (over 3 mm gross movement) motion parameters (*x, y, z*) + (yaw, pitch, roll) were added as nuisance regressors for deconvolution analysis. To control for multiple comparisons, a False Discovery Rate correction (Genovese et al., 2002) procedure was utilized on fMRI statistical maps using AFNI’s 3dFDR program with a *q*-value set to 0.05.

##### fMRI within-group analyses

We hypothesized that sedentary middle age adults would show more positive BOLD voxels in their right sensorimotor cortex (rM1S1), while young adults and physically active middle age adults would show a greater number of negative BOLD voxels in this region. To assess this hypothesis, we performed a within-group analysis of suprathreshold voxels (*q* < 0.05) in primary motor cortex. For this procedure, functional images were spatially smoothed using a 5-mm full-width half-maximum (FWHM) Gaussian filter to compensate for variability in structural and functional anatomy across participants. Anatomic and functional images were then interpolated to volumes with 2 mm^3^ voxels and converted to MNI152 standard space as implemented by AFNI. Estimates of hemodynamic response functions (HRF) were generated from a block regressor deconvolution procedure, as implemented by AFNI’s 3dDeconvolve (Ward, 2002), for each participant. We then summed the values of the impulse response function to get the area-under-the-curve (AUC) of each voxel. These values were then tested against baseline activity using AFNI’s 3dttest++.

##### fMRI between group analyses

Comparisons were made between groups of estimates of HRF derived from the above-described deconvolution analysis. Each MNI152 transformed voxel’s estimated M1 activity proportion was entered into a voxel-wise between-subjects *t*-test (using AFNI’s 3dttest++) for each pair-wise comparison of the four groups for each functional task.

#### Correlation analyses

##### Estimated VO_2_max with iSP and AUC fMRI data

This study was primarily interested in investigating the relationship of physical activity to neural correlates of aging-related changes in activity in the ipsilateral motor cortex. To test this relationship of physical fitness and fMRI amplitude change in BOLD response and the iSP, we completed a correlational analysis for: (a) all subjects and (b) within fitness groups. AUC measures were derived using the same M1 region of interest mask used for the impulse response analysis.

##### Motor performance measures with VO_2_max

To assess the relationship of physical fitness with respect to motor hand function, we also tested the correlation of our behavioral motor performance assessments with the estimated VO_2_max assessment. The behavioral measures included strength (hand grip, pinch grip), psychomotor speed (simple visuomotor reaction time, motor tapping), dexterity (9-hole pegboard, Purdue pegboard, coin-rotation task), and interhemispheric transfer (Poffenberger).

##### Motor performance measures with iSP and AUC fMRI data

We hypothesized a strong relationship between loss of interhemispheric inhibition with sedentary aging and subsequent loss of motor function. To test relationship, we correlated behavioral motor performance assessments (listed in above paragraph) with TMS and fMRI findings of aging-related change in iM1S1 activity.

## Results

### Behavioral measures

Behavioral data for the motor assessments are detailed in Table 5. Physically fit middle age adults showed better performance on measures of hand strength, dexterity, and psychomotor speed, as compared to their sedentary age cohort. There were no statistically significant differences in behavioral tests of motor performance between fit and sedentary younger adults. Respective of this, we collapsed this group to a “young adult” group consisting of *n* = 21 for contrast to sedentary and fit middle age adults.

**Table 5:** **Behavioral measures**.

	Sedentary middle age	Fit middle age	Younger
**HAND STRENGTH (PSI)**			
Hand grip left	63.47 (22.25)	75.5 (18.9)	63.1 (18.9)
Hand grip right	66.95 (21.5)	78.47 (19.1)	69.42 (20.9)
Pinch grip left	11.85 (2.97)^a^	14.23 (3.66)^a^	13.1 (3.91)
Pinch grip right	12.67 (3.03)^a^	15.4 (3.55)^a^	14.25 (3.5)
**DEXTERITY**			
9-Hole pegboard (s)	18.79 (3.03)^a^^,^^b^	16.21 (1.76)^a^	16.72 (1.94)^b^
Purdue (pegs)	15.85 (2.5)	16.23 (1.25)	16.47 (2.3)
Purdue (assembly)	9.93 (1.55)	9.68 (1.01)	10.28 (0.9)
Coin-rotation L (uni)	15.34 (3.37)	13.05 (2.18)	13.74 (3.3)
Coin-rotation R (uni)	14.99 (3.77)^b^	13.28 (2.89)	12.1 (2.7)^b^
Coin-rotation L (bi)	12.60 (2.3)	12.73 (1.4)	13.44 (2.96)
Coin-rotation R (bi)	12.95 (2.41)	13.16 (2.72)	12.18 (2.42)
Coin-rotation diff L	2.74 (2.03)^a^^,^^b^	0.31 (2.01)	0.29 (1.98)^b^
Coin-rotation diff R	2.03 (3.24)^a^^,^^b^	0.09 (1.83)	−0.04 (2.32)^b^
**PSYCHOMOTOR SPEED**			
Reaction time (ms)	289.2 (32.2)^b^	269.3 (32.3)	252.05 (27.8)^b^
Halstead finger tap	43.86 (8.7)^a^	52.29 (7.26)^a^	47.90 (8.7)
Poffenberger CUD (ms)	3.1 (0.5)	3.1 (0.9)	3.2 (0.3)

Coin rotation (uni) indicates unimanual coin-rotation score. Coin rotation (bi) indicates bimanual coin-rotation score. Coin-rotation diff (L/R) represents difference between unimanual and bimanual coin rotation task conditions (Left and right, respectively). Poffenberger CUD represents difference between crossed and uncrossed conditions. Cell values denote group means. Parentheses indicate standard deviation within cell. Student’s *t*-test contrast significance at *p* < 0.05 denoted by:

*^a^ For sedentary vs. fit middle age*.

*^b^ For sedentary vs. younger*.

### Mirror movement assessment

Mirror movements above EMG threshold (EMG signal in ipsilateral hand ≥20% of active pinch squeeze) were not present in any of the assessed individuals during TMS. No liminal mirror movements were detected during fMRI during the motor tapping condition.

### Transcranial magnetic stimulation

Presented in Table 6 are group data from TMS with associated *t*-tests and significance. There were no significant differences between fit and sedentary younger group on TMS measures, as such, we collapsed across these groups to compare a “young adult” group to the sedentary and fit middle age groups. Sedentary middle age adults had significantly shorter silent periods as compared to fit middle age and younger adults. LMT value did not significantly differ between any subject groups.

**Table 6:** **Transcranial magnetic stimulation measures**.

	Sedentary middle age	Fit middle age	Younger
iSP duration (ms)	43.9 (6.5)^a^^,^^b^	51.4 (7.4)^a^	50.14 (6.5)^b^
Lowest motor threshold (%)	46.3 (18.25)	41.4 (19.5)	40.5 (15.9)

Cell values denote group means. Parentheses indicate standard deviation within cell. Student’s *t*-test contrast significance at p < 0.05 denoted by:

*^a^ For sedentary vs. fit middle age*.

*^b^ For sedentary vs. younger*.

### Functional magnetic resonance imaging

#### Task-to-baseline comparisons

**Figure 3:**
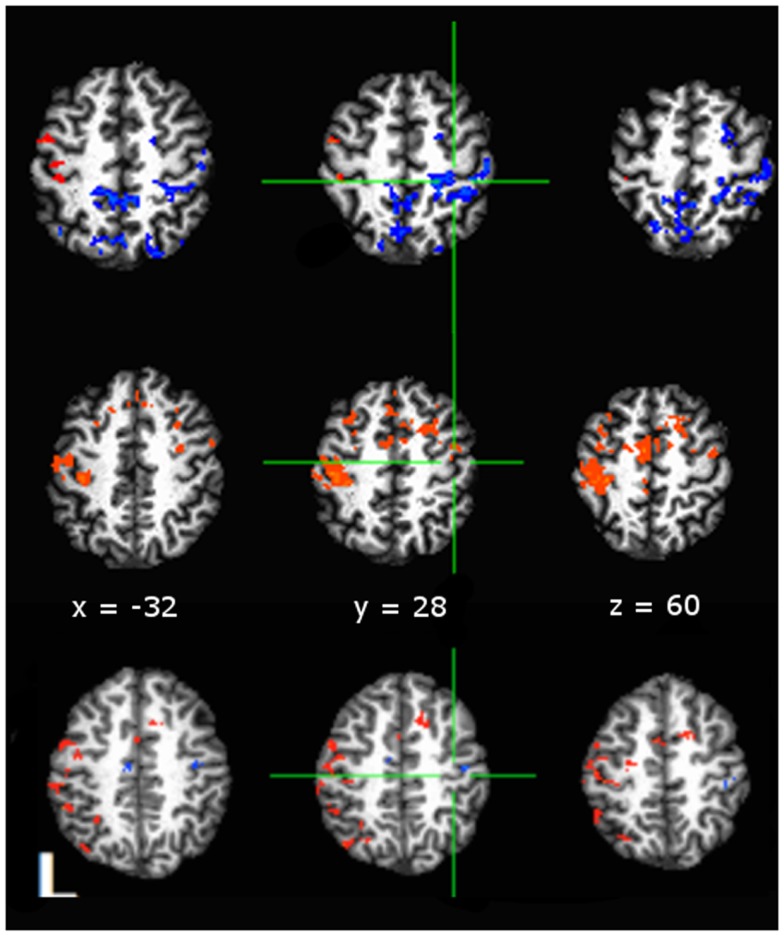
**Comparisons of area-under-curve (AUC) of the deconvolved hemodynamic response during performance of a tapping task taken against a null baseline condition with significance set to *p* < 0.05, False Discovery Rate corrected (Hues: orange indicates positive *t*-statistic, blue indicates negative *t*-statistic)**. Data shown for: top row, active middle age adults; middle row, sedentary middle age adults; bottom row, younger adults. Both middle age adults and young adults showed a negative to baseline pattern of activity in rM1, while sedentary middle age adults showed positive to baseline activity in rM1. Anatomical underlay is a skull-stripped standardized (MNI152) T1 image.

We were interested in the qualitative difference in response of the hemodynamic response (HDR) respective of grouping having hypothesized that cardiovascular fitness maintains negative BOLD in aging adults. Figure 3 presents comparisons of AUC values depicting whole-brain *t*-test comparisons (3dttest++) of activity to baseline within each grouping. Color-coding indicates direction of BOLD response with orange indicating positive AUC values relative to baseline and blue indicating negative AUC values. As shown in Figure 3 (middle row), physically fit middle age adults showed negative BOLD in right (ipsilateral) M1S1 cortex, as compared to sedentary middle age adults (Figure 3 – middle row) who showed positive BOLD in rM1S1. Younger adults showed negative BOLD in ipsilateral M1S1 regardless of group (Figure 3 – bottom row).

#### Group comparisons

**Figure 4:**
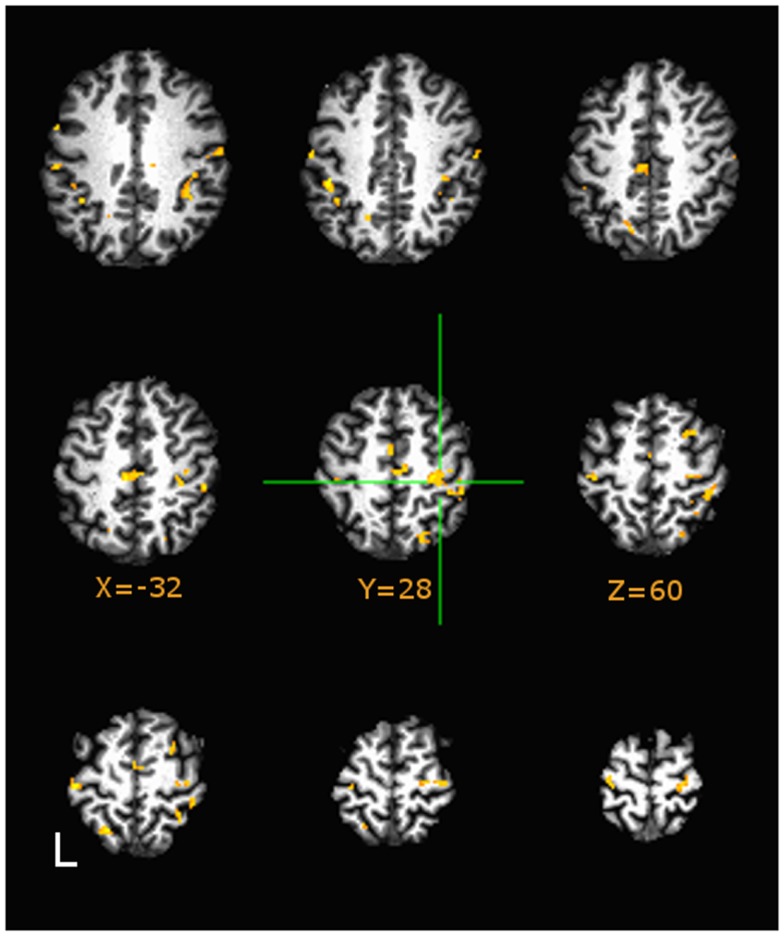
**Group differences in area-under-curve (AUC) group comparisons between Sedentary and Fit middle age adults on tapping task at *p* < 0.05, Image montage is in axial presentation of supra-callosal MNI152 registered slices separated by 6 mm**. False Discovery Rate (*q* < 0.05) corrected comparisons are presented in color (Hues: orange indicates more positive values in Sedentary adults, blue indicates vice-versa). Sedentary middle age adults showed significantly higher values of AUC (corresponding to positive BOLD) in right sensorimotor areas.

Direct group comparisons on AUC (3dttest++) of estimated HDR profiles (quantitatively described by the area-the-curve of the HDR) during motor tapping tasks are presented in Figure 4. Hue in the figure indicates minimum significance at *p* < 0.05, False Discovery Rate corrected. Figure 4 (in axial presentation of supra-callosal slices) indicates comparison of sedentary middle age against physically fit middle age adults (orange indicates higher AUC in sedentary). As compared to sedentary middle age adults, both physically active middle age adults and young adults evidenced significantly lower values of AUC relative to baseline in the hand knob of right motor cortex during the tapping task.

### Correlation analyses

#### Correlation analysis of VO_2_max estimate with iSP and AUC fMRI data

**Figure 5:**
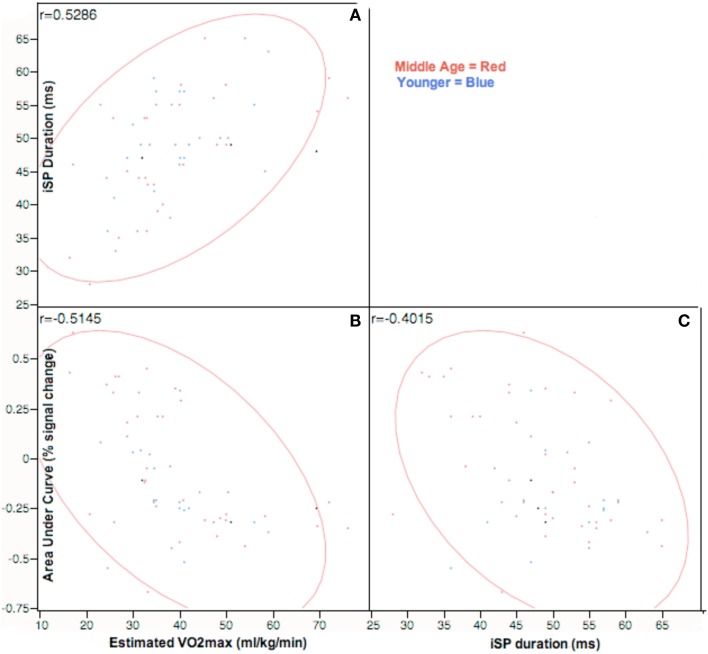
**Correlation analysis of VO_2_max assessments against ipsilateral silent period and area-under-curve values on tapping task at *p* < 0.05, Across participants, VO_2_max estimates are positively correlated with both iSP duration and negatively correlated with AUC estimates in rM1S1**. Right M1S1 AUC values are negatively correlated to duration of ipsilateral silent period. Correlations are represented in the top left corner for comparisons of: **(A)** iSP duration and estimated VO_2_max; **(B)** fMRI AUC of right M1S1 and VO_2_max; **(C)** iSP duration and fMRI AUC of right M1S1.

We hypothesized that physical fitness level would be correlated with measures of interhemispheric inhibition. Figure 5 presents correlational data across all participants between estimated VO_2_max against iSP and area under curve of right sensorimotor areas from fMRI across all participants. VO_2_max was strongly positively correlated with iSP and strongly negatively correlated with AUC of the right (ipsilateral) sensorimotor cortex. Within age-groupings, middle age adults showed a significantly positive correlation [*r*(36) = 0.56, *p* < 0.01] of VO_2_max with iSP duration and significantly negative correlation [*r*(36) = −0.60, *p* < 0.01] of VO_2_max with AUC of right sensorimotor cortex. Interestingly, within younger adults, there were no significant correlations between VO_2_max and either iSP duration or fMRI data.

As shown in Table 7, iSP duration was negatively correlated with AUC of the right (ipsilateral sensorimotor cortex) in middle age adults: *r*(36) = −0.46, *p* < 0.03. These measures did not show significant correlation in younger adults.

**Table 7:** **Significant pair-wise correlations of measures showing across middle age adults**.

Variable	Variable	Correlation	Count	Significant prob
fMRI_RM1S1	VO_2_max	−0.6147	38	<0.0001^a^
iSP duration	VO_2_max	0.5676	38	0.0002^a^
fMRI_RM1S1	Reaction time	0.507	38	0.0012^a^
iSP duration	fMRI_RM1S1	−0.4622	38	0.0035^a^
Halstead	VO_2_max	0.412	38	0.0102^a^
iSP duration	Reaction time	−0.376	38	0.02^a^
Reaction time	VO_2_max	−0.371	38	0.0218^a^
Halstead	9-hole	−0.3724	38	0.0213^a^
9-Hole	fMRI_RM1S1	0.3719	38	0.0215^a^
9-Hole	VO_2_max	−0.3692	38	0.0226^a^

*fMRI_RM1S1, fMRI area under curve of right sensorimotor cortex; iSP duration, duration of ipsilateral silent period; Halstead, Halstead finger tapping; 9-hole, 9-hole pegboard test*.

*^a^ Indicates significant Pearson correlation*.

In summary, across participants, physical fitness was significantly correlated with measures of interhemispheric inhibition. However, when we analyzed within each participant grouping, the results indicated that these effects were restricted to middle age adults.

#### Motor performance measures with VO_2_max

Across participants, there were significant correlations between estimated VO_2_max and the following motor assessments: Halstead finger tapping [*r*(57) = 0.39, *p* < 0.01]; 9-hole pegboard test [*r*(57) = −0.35, *p* < 0.01]; Simple Reaction Time [*r*(57) = −0.31, *p* = 0.02]. Within age groups, middle aged adults (Table 6) also showed significant correlations between VO_2_max and the same motor measures: Halstead [*r*(36) = 0.42, *p* < 0.01]; 9-Hole pegboard [*r*(36) = −0.36, *p* = 0.02]; Simple Reaction Time [*r*(36) = −0.37, *p* = 0.02]. No significant correlations between VO_2_max and motor performance metrics were evident across younger adults.

In summary, measures of physical fitness were significantly correlated with motor performance. However, these correlations were restricted to middle age groups underscoring that alteration of motor function respective of physical fitness is an aging-related phenomenon.

#### Motor performance measures with iSP and AUC fMRI data

Table 7 presents significant correlations in middle age adults of motor measures with both silent period and fMRI activity in M1S1. Dexterity (9-Hole pegboard) and psychomotor speed (Reaction time, finger tapping) showed significant correlations with both iSP and fMRI. In younger adults, a trend [*r*(19) = −0.31, *p* = 0.07] existed between silent period duration and reaction time. No other significant relationships between motor measures and TMS or fMRI were found within younger adults.

Importantly, the significant correlations in middle age adults provide a link between aging-related changes in interhemispheric inhibition and alteration of motor performance. Interestingly, though we had hypothesized this effect would be present regardless of age group; we did not find a relationship between level of interhemispheric inhibition and motor performance in younger adults. This, again, may indicate that the aging-related alteration of interhemispheric communication is the strong driver of behavioral change in motor performance.

## Discussion

The current study investigated the effects of physical fitness on interhemispheric inhibition and motor performance previously shown to differ across elderly and younger groups. The study extends the previous findings across the lifespan and shows that changes in interhemispheric activity occur within middle age (40–60), as well as in elderly (60+) populations (McGregor et al., 2011). Importantly, loss of interhemispheric inhibition appears to have deleterious effects on motor function. That is, sedentary middle age adults showed larger levels of positive BOLD activity in ipsilateral cortex and short iSPs with coincident decreased motor performance (psychomotor speed and dexterity). Further, the alterations of cortical activity and performance deficits respective of fitness level appear to be aging-related, as comparisons of physically fit and sedentary younger adults did not show such differences. To the best of our knowledge, this is the first study to show that sedentary aging, even in middle age, confers a loss of interhemispheric inhibition with negative effects on motor function, but cardiovascular fitness may mitigate these losses.

There is a growing body of evidence that has shown that the aging process is associated with altered communication in the primary motor cortices (Sale and Semmler, 2005; Riecker et al., 2006; Fujiyama et al., 2009, 2012; Langan et al., 2010; Davidson and Tremblay, 2013). The functional relevance of such changes to motor performance has been a matter of debate, however. The neurophysiological and imaging studies to date have bred essentially three lines of thought. The first of which is that aging-related alteration of patterns of cortical activity may be epiphenomenological and not interact with the function of the motor system. A wealth of evidence, however, has shown that alteration of motor function are associated with changes in levels interhemispheric inhibition (Talelli et al., 2008a,b; Giovannelli et al., 2009; Langan et al., 2010; Fling and Seidler, 2011; Fling et al., 2011a; McGregor et al., 2011, 2012a; Bernard and Seidler, 2012). Interestingly, the same interaction of function and alteration of interhemispheric inhibition has been reported in investigations of the sensorimotor cortex using median nerve stimulation during fMRI. In a series of studies, one lab has systematically shown that the levels of ipsilateral negative BOLD in primary sensory cortex (S1) are modified by varying the level of median nerve stimulation (Kastrup et al., 2008; Klingner et al., 2010, 2011). Moreover, this lab has shown evidence that the amount of ipsilateral S1 BOLD response during median nerve stimulation varies as a function of age with older adults (55+ years) showing lower levels of interhemispheric S1 inhibition (Gröschel et al., 2013).

A second interpretation of aging-related loss of inhibition in M1S1 involves the notion of aging-related cortical compensation. Largely driven by reports using fMRI, this view argues that increased bilateral positive BOLD of motor cortex in aging adults acts in a compensatory function to assist task completion (Mattay et al., 2002; Heuninckx et al., 2005; Wu and Hallett, 2005; Zimerman et al., in press). The critical assumption in this opinion is that the aging brain loses the capacity (via gray or white matter dysfunction) to complete tasks without the intervention of additional neural substrates. This, in turn, argues for a hierarchical pattern of activity across hemispheres whereby more difficult tasks require the intervention of the ipsilateral cortex. While related literature has shown some support for this hierarchical recruitment hypothesis (Hutchinson et al., 2002; Verstynen et al., 2005; Stippich et al., 2007), it is challenged by other studies that do not show increased bilaterality of M1 recruitment with greater task demands (Wu and Hallett, 2005; Riecker et al., 2006; McGregor et al., 2009; Van Impe et al., 2009). In large part, the idea of increased activation in ipsilateral cortex as a compensatory function is derived from models of alteration in cognitive functions more specific to the prefrontal cortex. Perhaps the most noteworthy model is the hemispheric asymmetry reduction in older adults (HAROLD) model posited by Cabeza (2002). However, work in the field has evolved to refine our understanding of aging-related changes in laterality of recruitment that is more domain-specific (e.g., the compensation related utilization of neural circuits – CRUNCH). However, the core assumption of compensation still remains.

The third interpretation of aging-related change in interhemispheric inhibition and motor function is that increased recruitment of the ipsilateral cortex during unimanual movements exacts a deleterious effect on motor performance. The current manuscript lends support to this, as sedentary middle age adults who had less interhemispheric inhibition during unimanual tasks performed worse on tests of psychomotor speed and dexterity. Further, as described in McGregor et al. (2012a), the engagement (pinch squeeze) of the ipsilateral motor cortex during a bimanual task (coin rotation), eliminates the dexterity advantage enjoyed by individuals with intact interhemispheric inhibition. It is likely that transcallosal communication during unimanual tasks is inhibitory in nature (Meyer et al., 1998; Stefanovic et al., 2004; Manson et al., 2006, 2008; Lenzi et al., 2007; Giovannelli et al., 2009; Llufriu et al., 2012) within healthy individuals and its reduction in older adults (and in instances of frank pathology) hinders proper task performance. There are numerous studies that have shown that increasing excitability in the contralateral motor cortex via anodal transcranial direct stimulation results in improved motor learning capacity and better motor performance (Hummel et al., 2010; Furuya et al., 2013; Saucedo Marquez et al., 2013; Zimerman et al., 2013). Corollary findings exist in different cognitive domains, as well. An interesting recent study combining tDCS and fMRI by Meinzer et al. (2013), investigated word production in aging and noted that the more likely older adults were to activate Broca’s homolog (right frontal operculum) in fMRI, the worse was their language performance. However, when anodal tDCS was applied to Broca’s area (left frontal operculum) in older adults, fMRI activity during word production remained lateralized to the left hemisphere and performance was markedly improved (Meinzer et al., 2013).

As the current study is one of the first to examine the functional implications of aging-related loss of interhemispheric inhibition on upper extremity motor performance using a multi-modality approach, additional research is required to explore the implications of these findings. It is important to state however, that the authors of this work do not intend to argue that aging-related change in patterns of activity in the primary motor cortex subsume all aspects of motor function be they unimanual or bimanual. Clearly, the alteration of functional networks of activity involving motor planning centers (SMA/PreSMA) and prefrontal executive areas is a driving factor in much of the changes reported in the current manuscript (Voelcker-Rehage et al., 2010; Fling and Seidler, 2011, 2012; Fling et al., 2011b; Bernard and Seidler, 2012). One structure of clear importance in the modulation of interhemispheric and network communication is the corpus callosum. Recent findings have pointed that alteration of the density of the corpus callosum is strongly implicated in the change of both resting state and functional connectivity of the motor cortices (Fling et al., 2011a; Bernard and Seidler, 2012). We approached callosal function in the current work using a crossed-uncrossed difference test (Poffenberger paradigm). Surprisingly, we found no differences between groups regardless of age or fitness level in contrast to Bernard and Seidler (2012). It may be that this assessment is not sensitive enough to differentiate functional change at the currently tested age ranges. Further work using more sensitive behavioral metrics of callosal function is needed to explore this critical anatomic structure in light of past and present findings.

Perhaps the most important finding within the current study is that even in middle age, sedentary aging confers a loss of interhemispheric inhibition with negative effects on motor function, but having higher levels of cardiovascular fitness may mitigate these losses. The current study extends and refines previous reports by our lab (McGregor et al., 2011, 2012a) and others (Marks et al., 2007; Voelcker-Rehage et al., 2010) that cardiovascular fitness is a strong driver of neuroplasticity in the motor system. A great deal of attention has been focused on the effects of cardiovascular fitness on the human neural system with respect to aging and cognition, and rightfully so (Colcombe et al., 2003, 2004a,b, 2006; Voelcker-Rehage et al., 2010, 2011; Erickson et al., 2011a,b; Prakash et al., 2011; Voss et al., 2011; Szabo et al., 2012). While it is, of course, impossible to dissociate cognition from motor control, the present findings indicate that physical fitness has a large impact on even very simple motor processes. This suggests that increased cardiovascular fitness over time may exert a core prophylactic effect to central nervous system changes that occur due to the aging process.

There are two notable findings involving the methodologies used within this study. The first of which is that the present study replicates previous findings (McGregor et al., 2011) of the strong relationship between BOLD activity in the ipsilateral motor cortex and the duration of the iSP. The relationship between neurophysiological measures of inhibition and BOLD imaging (negative BOLD in particular) has been an area of active study (Hayes and Huxtable, 2012; Muthukumaraswamy et al., 2012; Zeharia et al., 2012). Previous work has shown that areas of the motor cortex exhibiting negative BOLD during unimanual tasks have decreased blood flow using arterial spin labeling techniques (Stefanovic et al., 2004). However, the correspondence of negative BOLD in sensorimotor cortex to EMG or electrophysiology has only recently been explored (Yuan et al., 2011; Sarfeld et al., 2012). A recent study by Sarfeld et al. (2012) with younger adults using neuronavigated TMS tested the iSP in conjunction with the peak activity within the contralateral primary motor cortex. The results showed that the individuals with the highest peak contralateral BOLD activity showed the longest iSPs. The authors interpreted the results to indicate that the larger the inhibitory transcallosal transfer (due to increased BOLD signal in contralateral cortex) in these subjects was the likely driver of the longer silent period tested with TMS. (The authors did not report about negative BOLD response and used a unidirectional test to evaluate HDR.) The Sarfeld et al. (2012) findings relate to the current project in that individuals with longer iSPs also showed larger negative BOLD response in ipsilateral cortex. Interestingly, in the present study we did not see differences in contralateral motor cortex activity between groups. Further study is needed to investigate the interaction of cortical recruitment patterns in fMRI with electro and neurophysiological measures.

Secondly, the present study, we believe, is the first of its kind to differentiate both middle age and younger adults into fitness levels to assess the effects of physical fitness level on motor cortical function. Within our younger samples, we found no assessments (apart from BMI, fitness level, and reported activity level) that showed significant differences between fitness levels. This finding is perhaps limited due to the rather low sample size (*n* = 21), however, the results were highly systematic across TMS, imaging, and behavioral motor assessments. We interpret these results to support the contention that the alteration of interhemispheric communication reported in the current project is largely due to the aging process. However, a great deal of additional study is required to explore the effects of cardiovascular fitness across the lifespan.

There are some limitations that should be noted about the current work. First, we would like to point out that our physically fit middle age adults were well above the normative values (and even ACSM recommendations) for physical activity in this age group. Many of these individuals engaged in physical exercise as part of their profession (trainers, fitness instructors, exercise scientists). This was an intended outcome as we strived to study a clear stratification between the fitness levels of the two groups from the outset of the study. While we acknowledge that this may somewhat limit how accurately we can infer these findings to the general population, our present results indicate the strong effect of physical activity level on the motor system of middle aged adults and far outweigh the potential limitations. Secondly, the small sample size in younger adults and relatively high level of assessed fitness across all groups (e.g., sedentary young adults reported a VO_2_max of ∼30 ml/min/kg) may introduce floor effects with respect to differences in physical fitness level. With a larger and more diverse sample size, detection of motor and neurological functioning differences between fit and sedentary younger groups may be possible. Finally, cross-sectional studies such as the current one, while instructive, are not as convincing as a direct interventional approach. Future work should involve recruiting sedentary individuals into both a short and long-term exercise program to test the plasticity of patterns cortical activity in response to a regimented fitness intervention.

In conclusion, we believe the present work represents the first report using both fMRI and TMS to assess the relationship of physical activity, interhemispheric inhibition, and upper extremity motor performance across middle age adults. A major implication of the study is that the long-standing relationship between physical fitness and upper extremity motor performance (Spirduso, 1975) in aging is influenced by changes in interhemispheric communication within the cerebral cortex.

## Conflict of Interest Statement

The authors report no actual or potential conflicts of interest. The contents do not represent the views of the Department of Veterans Affairs or the United States Government. This work was supported by a Department of Veteran Affairs (VA) Rehabilitation R&D Center of Excellence #F2182C, Career Development Awards Level-1 (Keith M. McGregor; Joe R. Nocera), and Senior Research Career Scientist (Bruce Crosson: #B6364L) awards.

## References

[B1] Allison J. D. Meador K. J. Loring D. W. Figueroa R. E. Wright J. C. (2000). Functional MRI cerebral activation and deactivation during finger movement. Neurology 54, 135–142 10.1212/WNL.54.1.135 10636139

[B2] Bernard J. A. Seidler R. D. (2012). Evidence for motor cortex dedifferentiation in older adults. Neurobiol. Aging 33, 1890–1899 10.1016/j.neurobiolaging.2011.06.021 21813213 PMC3391352

[B3] Bernard J. A. Taylor S. F. Seidler R. D. (2011). Handedness, dexterity, and motor cortical representations. J. Neurophysiol. 105, 88–99 10.1152/jn.00512.2010 20943944

[B4] Cabeza R. (2002). Hemispheric asymmetry reduction in older adults: the HAROLD model. Psychol. Aging 17, 85–100 10.1037/0882-7974.17.1.85 11931290

[B5] Christou E. A. (2011). Aging and variability of voluntary contractions. Exerc. Sport Sci. Rev. 39, 77–84 10.1097/JES.0b013e31820b85ab 21206281 PMC3631580

[B6] Colcombe S. J. Erickson K. I. Raz N. Webb A. G. Cohen N. J. McAuley E. (2003). Aerobic fitness reduces brain tissue loss in aging humans. J. Gerontol. A Biol. Sci. Med. Sci. 58, 176–180 10.1093/gerona/58.2.M176 12586857

[B7] Colcombe S. J. Erickson K. I. Scalf P. E. Kim J. S. Prakash R. McAuley E. (2006). Aerobic exercise training increases brain volume in aging humans. J. Gerontol. A Biol. Sci. Med. Sci. 61, 1166–1170 10.1093/gerona/61.11.1166 17167157

[B8] Colcombe S. J. Kramer A. F. Erickson K. I. Scalf P. McAuley E. Cohen N. J. (2004a). Cardiovascular fitness, cortical plasticity, and aging. Proc. Natl. Acad. Sci. U.S.A. 101, 3316–3321 10.1073/pnas.0400266101 14978288 PMC373255

[B9] Colcombe S. J. Kramer A. F. McAuley E. Erickson K. I. Scalf P. (2004b). Neurocognitive aging and cardiovascular fitness: recent findings and future directions. J. Mol. Neurosci. 24, 9–14 10.1385/JMN:24:1:009 15314244

[B10] Cole K. J. Rotella D. L. Harper J. G. (1998). Tactile impairments cannot explain the effect of age on a grasp and lift task. Exp. Brain Res. 121, 263–269 10.1007/s002210050459 9746132

[B11] Cox R. W. (1996). AFNI: software for analysis and visualization of functional magnetic resonance neuroimages. Comput. Biomed. Res. 29, 162–173 10.1006/cbmr.1996.0014 8812068

[B12] Davidson T. Tremblay F. (2013). Age and hemispheric differences in transcallosal inhibition between motor cortices: an ispsilateral silent period study. BMC Neurosci. 14:62 10.1186/1471-2202-14-62 23800346 PMC3695846

[B13] Erickson K. I. Prakash R. S. Voss M. W. Chaddock L. Heo S. McLaren M. (2011a). Brain-derived neurotrophic factor is associated with age-related decline in hippocampal volume. J. Neurosci. 30, 5368–5375 10.1523/JNEUROSCI.6251-09.2010 20392958 PMC3069644

[B14] Erickson K. I. Voss M. W. Prakash R. S. Basak C. Szabo A. Chaddock L. (2011b). Exercise training increases size of hippocampus and improves memory. Proc. Natl. Acad. Sci U.S.A. 108, 3017–3022 10.1073/pnas.1015950108 21282661 PMC3041121

[B15] Fling B. W. Benson B. L. Seidler R. D. (2011a). Transcallosal sensorimotor fiber tract structure-function relationships. Hum. Brain Mapp. 34, 384–395 10.1002/hbm.21437 22042512 PMC3271163

[B16] Fling B. W. Peltier S. J. Bo J. Welsh R. C. Seidler R. D. (2011b). Age differences in interhemispheric interactions: callosal structure, physiological function, and behavior. Front. Neurosci. 5:38 10.3389/fnins.2011.00038 21519384 PMC3077973

[B17] Fling B. W. Seidler R. D. (2011). Fundamental differences in callosal structure, neurophysiologic function, and bimanual control in young and older adults. Cereb. Cortex 22, 2643–2652 10.1093/cercor/bhr349 22166764 PMC3464417

[B18] Fling B. W. Seidler R. D. (2012). Task-dependent effects of interhemispheric inhibition on motor control. Behav. Brain Res. 226, 211–217 10.1016/j.bbr.2011.09.018 21944939 PMC3208314

[B19] Fujiyama H. Garry M. I. Levin O. Swinnen S. P. Summers J. J. (2009). Age-related differences in inhibitory processes during interlimb coordination. Brain Res. 1262, 38–47 10.1016/j.brainres.2009.01.023 19368842

[B20] Fujiyama H. Hinder M. R. Schmidt M. W. Garry M. I. Summers J. J. (2012). Age-related differences in corticospinal excitability and inhibition during coordination of upper and lower limbs. Neurobiol. Aging 33, e1–e14 10.1016/j.neurobiolaging.2011.12.019 22257984

[B21] Furuya S. Nitsche M. A. Paulus W. Altenmuller E. (2013). Early optimization in finger dexterity of skilled pianists: implication of transcranial stimulation. BMC Neurosci. 14:35 10.1186/1471-2202-14-35 23496918 PMC3616936

[B22] Garatachea N. Cavalcanti E. Garcia-Lopez D. Gonzalez-Gallego J. de Paz J. A. (2007). Estimation of energy expenditure in healthy adults from the YMCA submaximal cycle ergometer test. Eval. Health Prof. 30, 138–149 10.1177/0163278707300628 17476027

[B23] Garvey M. A. Ziemann U. Becker D. A. Barker C. A. Bartko J. J. (2001). New graphical method to measure silent periods evoked by transcranial magnetic stimulation. Clin. Neurophysiol. 112, 1451–1460 10.1016/S1388-2457(01)00581-8 11459685

[B24] Genovese C. R. Lazar N. A. Nichols T. (2002). Thresholding of statistical maps in functional neuroimaging using the false discovery rate. Neuroimage 15, 870–878 10.1006/nimg.2001.1037 11906227

[B25] Giovannelli F. Borgheresi A. Balestrieri F. Zaccara G. Viggiano M. P. Cincotta M. (2009). Modulation of interhemispheric inhibition by volitional motor activity: an ipsilateral silent period study. J. Physiol. 587, 5393–5410 10.1113/jphysiol.2009.175885 19770195 PMC2793872

[B26] Gordon B. A. Rykhlevskaia E. I. Brumback C. R. Lee Y. Elavsky S. Konopack J. F. (2008). Neuroanatomical correlates of aging, cardiopulmonary fitness level, and education. Psychophysiology 45, 825–838 10.1111/j.1469-8986.2008.00676.x 18627534 PMC5287394

[B27] Gröschel S. Sohns J. M. Schmidt-Samoa C. Baudewig J. Becker L. Dechent P. (2013). Effects of age on negative BOLD signal changes in the primary somatosensory cortex. NeuroImage 71, 10–18 10.1016/j.neuroimage.2012.12.039 23296182

[B28] Hanna-Pladdy B. Mendoza J. E. Apostolos G. T. Heilman K. M. (2002). Lateralised motor control: hemispheric damage and the loss of deftness. J. Neurol. Neurosurg. Psychiatry 73, 574–577 10.1136/jnnp.73.5.574 12397154 PMC1738143

[B29] Hayes D. J. Huxtable A. G. (2012). Interpreting deactivations in neuroimaging. Front. Psychol. 3:27 10.3389/fpsyg.2012.00027 22347207 PMC3273719

[B30] Hermsdorfer J. Danek A. Winter T. Marquardt C. Mai N. (1995). Persistent mirror movements: force and timing of “mirroring” are task-dependent. Exp. Brain Res. 104, 126–134 10.1007/BF00229862 7621931

[B31] Heuninckx S. Wenderoth N. Debaere F. Peeters R. Swinnen S. P. (2005). Neural basis of aging: the penetration of cognition into action control. J. Neurosci. 25, 6787–6796 10.1523/JNEUROSCI.1263-05.2005 16033888 PMC6725362

[B32] Hlushchuk Y. Hari R. (2006). Transient suppression of ipsilateral primary somatosensory cortex during tactile finger stimulation. J. Neurosci. 26, 5819–5824 10.1523/JNEUROSCI.5536-05.2006 16723540 PMC6675271

[B33] Hummel F. C. Heise K. Celnik P. Floel A. Gerloff C. Cohen L. G. (2010). Facilitating skilled right hand motor function in older subjects by anodal polarization over the left primary motor cortex. Neurobiol. Aging 31, 2160–2168 10.1016/j.neurobiolaging.2008.12.008 19201066 PMC2995492

[B34] Hutchinson S. Kobayashi M. Horkan C. M. Pascual-Leone A. Alexander M. P. Schlaug G. (2002). Age-related differences in movement representation. Neuroimage 17, 1720–1728 10.1006/nimg.2002.1309 12498746

[B35] Jancke L. Shah N. J. Peters M. (2000). Cortical activations in primary and secondary motor areas for complex bimanual movements in professional pianists. Brain Res. Cogn. Brain Res. 10, 177–183 10.1016/S0926-6410(00)00028-8 10978706

[B36] Kastrup A. Baudewig J. Schnaudigel S. Huonker R. Becker L. Sohns J. M. (2008). Behavioral correlates of negative BOLD signal changes in the primary somatosensory cortex. Neuroimage 41, 1364–1371 10.1016/j.neuroimage.2008.03.049 18495495

[B37] Kleim J. A. Kleim E. D. Cramer S. C. (2007). Systematic assessment of training-induced changes in corticospinal output to hand using frameless stereotaxic transcranial magnetic stimulation. Nat. Protoc. 2, 1675–1684 10.1038/nprot.2007.206 17641632

[B38] Klingner C. M. Hasler C. Brodoehl S. Witte O. W. (2010). Dependence of the negative BOLD response on somatosensory stimulus intensity. Neuroimage 53, 189–195 10.1016/j.neuroimage.2010.05.087 20538064

[B39] Klingner C. M. Huonker R. Flemming S. Hasler C. Brodoehl S. Preul C. (2011). Functional deactivations: multiple ipsilateral brain areas engaged in the processing of somatosensory information. Hum. Brain Mapp. 32, 127–140 10.1002/hbm.21006 21157879 PMC6870510

[B40] Krampe R. T. Engbert R. Kliegl R. (2002). The effects of expertise and age on rhythm production: adaptations to timing and sequencing constraints. Brain Cogn. 48, 179–194 10.1006/brcg.2001.1312 11812041

[B41] Langan J. Peltier S. J. Bo J. Fling B. W. Welsh R. C. Seidler R. D. (2010). Functional implications of age differences in motor system connectivity. Front. Syst. Neurosci. 4:17 10.3389/fnsys.2010.00017 20589101 PMC2893009

[B42] Latash M. L. Zatsiorsky V. M. (2009). Multi-finger prehension: control of a redundant mechanical system. Adv. Exp. Med. Biol. 629, 597–618 10.1007/978-0-387-77064-2_32 19227523

[B43] Lenzi D. Conte A. Mainero C. Frasca V. Fubelli F. Totaro P. (2007). Effect of corpus callosum damage on ipsilateral motor activation in patients with multiple sclerosis: a functional and anatomical study. Hum. Brain Mapp. 28, 636–644 10.1002/hbm.20305 17080438 PMC6871400

[B44] Llufriu S. Blanco Y. Martinez-Heras E. Casanova-Molla J. Gabilondo I. Sepulveda M. (2012). Influence of corpus callosum damage on cognition and physical disability in multiple sclerosis: a multimodal study. PLoS ONE 7:e37167 10.1371/journal.pone.0037167 22606347 PMC3351399

[B45] Manson S. C. Palace J. Frank J. A. Matthews P. M. (2006). Loss of interhemispheric inhibition in patients with multiple sclerosis is related to corpus callosum atrophy. Exp. Brain Res. 174, 728–733 10.1007/s00221-006-0517-4 16944115

[B46] Manson S. C. Wegner C. Filippi M. Barkhof F. Beckmann C. Ciccarelli O. (2008). Impairment of movement-associated brain deactivation in multiple sclerosis: further evidence for a functional pathology of interhemispheric neuronal inhibition. Exp. Brain Res. 187, 25–31 10.1007/s00221-008-1276-1 18236036 PMC2414440

[B47] Marks B. L. Madden D. J. Bucur B. Provenzale J. M. White L. E. Cabeza R. (2007). Role of aerobic fitness and aging on cerebral white matter integrity. Ann. N. Y. Acad. Sci. 1097, 171–174 10.1196/annals.1379.022 17413020

[B48] Marzi C. A. (1999). The Poffenberger paradigm: a first, simple, behavioural tool to study interhemispheric transmission in humans. Brain Res. Bull. 50, 421–422 10.1016/S0361-9230(99)00174-4 10643464

[B49] Mattay V. S. Fera F. Tessitore A. Hariri A. R. Das S. Callicott J. H. (2002). Neurophysiological correlates of age-related changes in human motor function. Neurology 58, 630–635 10.1212/WNL.58.4.630 11865144

[B50] McGregor K. Craggs J. Benjamin M. Crosson B. White K. (2009). Age-related changes in motor control during unimanual movements. Brain Imaging Behav. 3, 317–331 10.1007/s11682-009-9074-324005766

[B51] McGregor K. Heilman K. Nocera J. Patten C. Manini T. Crosson B. (2012a). Aging, aerobic activity and interhemispheric communication. Brain Sci. 2, 634–648 10.3390/brainsci2040634 PMC4061818 24961264

[B52] McGregor K. M. Carpenter H. Kleim E. Sudhyadhom A. White K. D. Butler A. J. (2012b). Motor map reliability and aging: a TMS/fMRI study. Exp. Brain Res. 219, 97–106 10.1007/s00221-012-3070-3 22466408

[B53] McGregor K. M. Zlatar Z. Kleim E. Sudhyadhom A. Bauer A. Phan S. (2011). Physical activity and neural correlates of aging: a combined TMS/fMRI study. Behav. Brain Res. 222, 158–168 10.1016/j.bbr.2011.03.042 21440574 PMC3713467

[B54] Meinzer M. Lindenberg R. Antonenko D. Flaisch T. Floel A. (2013). Anodal transcranial direct current stimulation temporarily reverses age-associated cognitive decline and functional brain activity changes. J. Neurosci. 33, 12470–12478 10.1523/JNEUROSCI.5743-12.2013 23884951 PMC6618670

[B55] Mendoza J. E. Apostolos G. T. Humphreys J. D. Hanna-Pladdy B. O’Bryant S. E. (2009). Coin rotation task (CRT): a new test of motor dexterity. Arch. Clin. Neuropsychol. 24, 287–292 10.1093/arclin/acp030 19592523

[B56] Meyer B. U. Röricht S. Gräfin von Einsiedel H. Kruggel F. Weindl A. (1995) Inhibitory and excitatory interhemispheric transfers between motor cortical areas in normal humans and patients with abnormalities of the corpus callosum. Brain 118(Pt 2), 429–440 10.1093/brain/118.2.429 7735884

[B57] Meyer B. U. Roricht S. Woiciechowsky C. (1998). Topography of fibers in the human corpus callosum mediating interhemispheric inhibition between the motor cortices. Ann. Neurol. 43, 360–369 10.1002/ana.410430314 9506553

[B58] Muthukumaraswamy S. D. Evans C. J. Edden R. A. Wise R. G. Singh K. D. (2012). Individual variability in the shape and amplitude of the BOLD-HRF correlates with endogenous GABAergic inhibition. Hum. Brain Mapp. 33, 455–465 10.1002/hbm.21223 21416560 PMC3374935

[B59] Naccarato M. Calautti C. Jones P. S. Day D. J. Carpenter T. A. Baron J. C. (2006). Does healthy aging affect the hemispheric activation balance during paced index-to-thumb opposition task? An fMRI study. Neuroimage 32, 1250–1256 10.1016/j.neuroimage.2006.05.003 16806984

[B60] Newton J. M. Sunderland A. Gowland P. A. (2005). fMRI signal decreases in ipsilateral primary motor cortex during unilateral hand movements are related to duration and side of movement. Neuroimage 24, 1080–1087 10.1016/j.neuroimage.2004.10.003 15670685

[B61] Oldfield R. C. (1971). The assessment and analysis of handedness: the Edinburgh inventory. Neuropsychologia 9, 97–113 10.1016/0028-3932(71)90067-4 5146491

[B62] Petitjean M. Ko J. Y. (2012). An age-related change in the ipsilateral silent period of a small hand muscle. Clin. Neurophysiol. 124, 346–353 10.1016/j.clinph.2012.07.006 22883478

[B63] Prakash R. S. Voss M. W. Erickson K. I. Lewis J. M. Chaddock L. Malkowski E. (2011). Cardiorespiratory fitness and attentional control in the aging brain. Front. Hum. Neurosci. 4:229 10.3389/fnhum.2010.00229 21267428 PMC3024830

[B64] Rao S. M. Binder J. R. Bandettini P. A. Hammeke T. A. Yetkin F. Z. Jesmanowicz A. (1993). Functional magnetic resonance imaging of complex human movements. Neurology 43, 2311–2318 10.1212/WNL.43.11.2311 8232948

[B65] Riecker A. Groschel K. Ackermann H. Steinbrink C. Witte O. Kastrup A. (2006). Functional significance of age-related differences in motor activation patterns. Neuroimage 32, 1345–1354 10.1016/j.neuroimage.2006.05.021 16798017

[B66] Saad Z. S. Glen D. R. Chen G. Beauchamp M. S. Desai R. Cox R. W. (2009). A new method for improving functional-to-structural MRI alignment using local Pearson correlation. Neuroimage 44, 839–848 10.1016/j.neuroimage.2008.09.037 18976717 PMC2649831

[B67] Sale M. V. Semmler J. G. (2005). Age-related differences in corticospinal control during functional isometric contractions in left and right hands. J. Appl. Physiol. 99, 1483–1493 10.1152/japplphysiol.00371.2005 15947031

[B68] Sarfeld A. S. Diekhoff S. Wang L. E. Liuzzi G. Uludag K. Eickhoff S. B. (2012). Convergence of human brain mapping tools: neuronavigated TMS parameters and fMRI activity in the hand motor area. Hum. Brain Mapp. 33, 1107–1123 10.1002/hbm.21272 21520346 PMC6870141

[B69] Saucedo Marquez C. M. Zhang X. Swinnen S. P. Meesen R. Wenderoth N. (2013). Task-specific effect of transcranial direct current stimulation on motor learning. Front. Hum. Neurosci. 7:333 10.3389/fnhum.2013.00333 23847505 PMC3696911

[B70] Schulte T. Sullivan E. V. Muller-Oehring E. M. Adalsteinsson E. Pfefferbaum A. (2005). Corpus callosal microstructural integrity influences interhemispheric processing: a diffusion tensor imaging study. Cereb. Cortex 15, 1384–1392 10.1093/cercor/bhi020 15635059

[B71] Seidler R. D. Bernard J. A. Burutolu T. B. Fling B. W. Gordon M. T. Gwin J. T. (2010). Motor control and aging: links to age-related brain structural, functional, and biochemical effects. Neurosci. Biobehav. Rev. 34, 721–733 10.1016/j.neubiorev.2009.10.005 19850077 PMC2838968

[B72] Spirduso W. W. (1975). Reaction and movement time as a function of age and physical activity level. J. Gerontol. 30, 435–440 10.1093/geronj/30.4.435 1141674

[B73] Spirduso W. W. (1980). Physical fitness, aging, and psychomotor speed: a review. J. Gerontol. 35, 850–865 10.1093/geronj/35.6.850 7002994

[B74] Spirduso W. W. MacRae H. H. MacRae P. G. Prewitt J. Osborne L. (1988). Exercise effects on aged motor function. Ann. N. Y. Acad. Sci. 515, 363–375 10.1111/j.1749-6632.1988.tb33010.x 3364895

[B75] Stefanovic B. Warnking J. M. Pike G. B. (2004). Hemodynamic and metabolic responses to neuronal inhibition. Neuroimage 22, 771–778 10.1016/j.neuroimage.2004.01.036 15193606

[B76] Stippich C. Blatow M. Durst A. Dreyhaupt J. Sartor K. (2007). Global activation of primary motor cortex during voluntary movements in man. Neuroimage 34, 1227–1237 10.1016/j.neuroimage.2006.08.046 17137794

[B77] Szabo A. N. Bangert A. S. Reuter-Lorenz P. A. Seidler R. D. (2012). Physical activity is related to timing performance in older adults. Neuropsychol. Dev. Cogn. B Aging Neuropsychol. Cogn. 20, 356–369 10.1080/13825585.2012.715625 22917438 PMC3528826

[B78] Talelli P. Ewas A. Waddingham W. Rothwell J. C. Ward N. S. (2008a). Neural correlates of age-related changes in cortical neurophysiology. Neuroimage 40, 1772–1781 10.1016/j.neuroimage.2008.01.039 18329904 PMC3715371

[B79] Talelli P. Waddingham W. Ewas A. Rothwell J. C. Ward N. S. (2008b). The effect of age on task-related modulation of interhemispheric balance. Exp. Brain Res. 186, 59–66 10.1007/s00221-007-1205-8 18040671 PMC2257995

[B80] Van Impe A. Coxon J. P. Goble D. J. Wenderoth N. Swinnen S. P. (2009). Ipsilateral coordination at preferred rate: effects of age, body side and task complexity. Neuroimage 47, 1854–1862 10.1016/j.neuroimage.2009.06.027 19539766

[B81] Verstynen T. Diedrichsen J. Albert N. Aparicio P. Ivry R. B. (2005). Ipsilateral motor cortex activity during unimanual hand movements relates to task complexity. J. Neurophysiol. 93, 1209–1222 10.1152/jn.00720.2004 15525809

[B82] Verstynen T. Spencer R. Stinear C. M. Konkle T. Diedrichsen J. Byblow W. D. (2007). Ipsilateral corticospinal projections do not predict congenital mirror movements: a case report. Neuropsychologia 45, 844–852 10.1016/j.neuropsychologia.2006.08.019 17023008 PMC2275211

[B83] Voelcker-Rehage C. Godde B. Staudinger U. M. (2010). Physical and motor fitness are both related to cognition in old age. Eur. J. Neurosci. 31, 167–176 10.1111/j.1460-9568.2009.07014.x 20092563

[B84] Voelcker-Rehage C. Godde B. Staudinger U. M. (2011). Cardiovascular and coordination training differentially improve cognitive performance and neural processing in older adults. Front. Hum. Neurosci. 5:26 10.3389/fnhum.2011.00026 21441997 PMC3062100

[B85] Voss M. W. Prakash R. S. Erickson K. I. Basak C. Chaddock L. Kim J. S. (2011). Plasticity of brain networks in a randomized intervention trial of exercise training in older adults. Front. Aging Neurosci. 2:32. 10.3389/fnagi.2010.00032 20890449 PMC2947936

[B86] Ward B. D. (2002). Deconvolution analysis of fMRI time series data. Available at: http://afni.nimh.nih.gov/pub/dist/doc/program_help/3dDeconvolve.html

[B87] Ward N. S. Swayne O. B. Newton J. M. (2008). Age-dependent changes in the neural correlates of force modulation: an fMRI study. Neurobiol. Aging 29, 1434–1446 10.1016/j.neurobiolaging.2007.04.017 17566608 PMC2568861

[B88] Wu T. Hallett M. (2005). The influence of normal human ageing on automatic movements. J. Physiol. 562, 605–615 10.1113/jphysiol.2004.076042 15513939 PMC1665504

[B89] Yuan H. Perdoni C. Yang L. He B. (2011). Differential electrophysiological coupling for positive and negative BOLD responses during unilateral hand movements. J. Neurosci. 31, 9585–9593 10.1523/JNEUROSCI.5312-10.2011 21715623 PMC3142625

[B90] Zeharia N. Hertz U. Flash T. Amedi A. (2012). Negative blood oxygenation level dependent homunculus and somatotopic information in primary motor cortex and supplementary motor area. Proc. Natl. Acad. Sci. U.S.A. 109, 18565–18570 10.1073/pnas.1119125109 23086164 PMC3494917

[B91] Zimerman M. Heise K. F. Gerloff C. Cohen L. G. Hummel F. C. (in press). Disrupting the ipsilateral motor cortex interferes with training of a complex motor task in older adults. Cereb. Cortex. 10.1093/cercor/bhs385 23242199

[B92] Zimerman M. Nitsch M. Giraux P. Gerloff C. Cohen L. G. Hummel F. C. (2013). Neuroenhancement of the aging brain: restoring skill acquisition in old subjects. Ann. Neurol. 73, 10–15 10.1002/ana.23761 23225625 PMC4880032

